# Pharmacological clearance of senescent cells improves survival and recovery in aged mice following acute myocardial infarction

**DOI:** 10.1111/acel.12945

**Published:** 2019-03-28

**Authors:** Anna Walaszczyk, Emily Dookun, Rachael Redgrave, Simon Tual‐Chalot, Stella Victorelli, Ioakim Spyridopoulos, Andrew Owens, Helen M. Arthur, João F. Passos, Gavin D. Richardson

**Affiliations:** ^1^ Cardiovascular Research Centre, Institute of Genetic Medicine Newcastle University Newcastle upon Tyne UK; ^2^ Institute for Cell and Molecular Biosciences Newcastle University Newcastle upon Tyne UK; ^3^ Department of Physiology and Biomedical Engineering Mayo Clinic Rochester Minnesota

**Keywords:** aging, cardiac, myocardial infarction, senescence, senolytics, survival

## Abstract

Cardiovascular disease is the leading cause of death in individuals over 60 years old. Aging is associated with an increased prevalence of coronary artery disease and a poorer prognosis following acute myocardial infarction (MI). With age, senescent cells accumulate in tissues, including the heart, and contribute to age‐related pathologies. However, the role of senescence in recovery following MI has not been investigated. In this study, we demonstrate that treatment of aged mice with the senolytic drug, navitoclax, eliminates senescent cardiomyocytes and attenuates profibrotic protein expression in aged mice. Importantly, clearance of senescent cells improved myocardial remodelling and diastolic function as well as overall survival following MI. These data provide proof‐of‐concept evidence that senescent cells are major contributors to impaired function and increased mortality following MI and that senolytics are a potential new therapeutic avenue for MI.

## 1 INTRODUCTION, RESULTS, DISCUSSION

Cardiovascular disease (CVD) is a prevalent disease in the older population. The incidence of atherosclerotic coronary artery disease (CAD) increases with age and is present in over 50% of people over 60 years old (North & Sinclair, 2012). As such, older patients have a higher incidence of MI, which is accompanied by increased morbidity and mortality (Mehta et al., 2001).

Cellular senescence is classically defined as the irreversible cell cycle arrest of somatic cells. While senescence can act as a potent antitumour mechanism, recent studies have shown that senescent cells accumulate in several tissues with age where they contribute to age‐dependent tissue dysfunction and several age‐related diseases (de Magalhaes & Passos, 2018; Kirkland & Tchkonia, 2017). Senescent cells are thought to contribute to aging via a pro‐oxidant phenotype (Passos et al., 2010) and the secretion of a senescence‐associated secretory phenotype (SASP) (Coppe et al., 2008), which is pro‐inflammatory, profibrotic and can also promote senescence in surrounding cells (Nelson et al., 2012).

Senescence has been shown to occur in the heart during aging (Anderson et al., 2019) and contributes to the pathophysiology of a number of CVDs, as clearance of senescent cells in aged and atherosclerotic mice using both genetic and pharmacological approaches improves vascular and myocardial function and attenuates age‐dependent remodelling (Anderson et al., 2019; Roos et al., 2016; Zhu et al., 2015). However, the impact of senescent cells in MI has not been investigated thus far. In this study, we hypothesise that senescent cells contribute to the poor prognosis and survival of aged individuals following MI.

Previously we found that in addition to clearing senescent cells, navitoclax treatment reduced fibrosis and cardiomyocyte (CM) hypertrophy in aged mice (Anderson et al., 2019) and considered that these beneficial effects may help to improve outcomes in aged mice following MI. We therefore performed a more detailed longitudinal study to examine this possibility and to explore potential mechanisms (detailed methods are included in Appendix S1).

**Figure 1:**
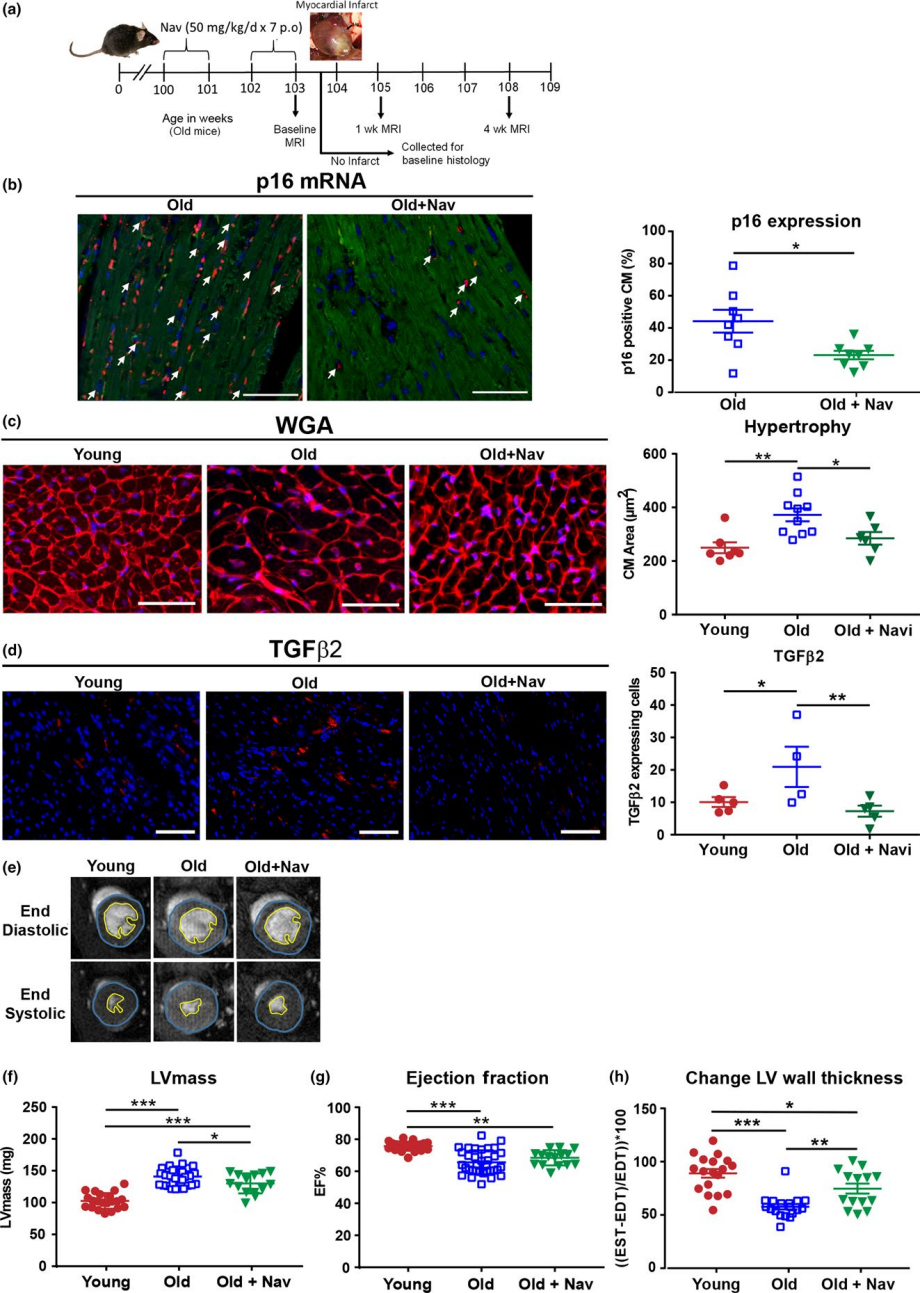
Aged mice display increased CM senescence, CM hypertrophy, increased TGFβ2 expression and functional characteristics of myocardial aging, which are attenuated by navitoclax treatment. (a) Experimental design. (b) Percentage p16^+^ CMs by RNA in situ hybridization. Arrow indicates p16 expressing CMs (p16 red, troponin‐C green, DAPI blue), *n* = 8 per group. Scale bars = 50 µm. (c) WGA staining and quantification of CM cross‐sectional area, *n* = 6–10 per group. (d) TGFβ2 protein expression, *n* = 4–5 per group. (e) Examples of individual short axis cine‐MR images. Analysis of (f) left ventricular mass and (g) ejection fraction. (h) % change in wall thickness, *n* = 14–34 per group. For c and d, scale bars = 100 µm. Data are mean ± *SEM*, ***p < 0.001; **p < 0.01; * *p* < 0.05 using Student's *t* test or one‐way ANOVA

Histological analysis was performed on a cohort of noninfarcted mice, to assess the baseline effects of navitoclax treatment (50 mg kg^−1^ day^−1^ in the regime shown in Figure 1a). In addition to decreasing CM hypertrophy, treatment reduced markers of CM senescence, indicating clearance of senescent cells from the hearts of treated aged mice (Figure 1b–c). Furthermore, we found a significant reduction in expression of profibrotic TGFβ2 (Figure 1d), which we previously identified as a key component of CM SASP (Anderson et al., 2019). Functionally, navitoclax treatment significantly reduced the age‐dependent increase in left ventricular (LV) mass (Figure 1f) but did not impact on ejection fraction (EF) (Figure 1g). Aged mice also exhibited a decrease in the percentage change in diastole versus end systole LV wall thickness, indicating an increased LV rigidity compared with young animals, which was also partly rescued by navitoclax treatment (Figure 1h). Clinically, increased ventricle stiffness is related to fibrosis and hypertrophy during aging, is symptomatic of diastolic dysfunction and is observed in heart failure with preserved ejection fraction patients (Borlaug, 2014).

To investigate whether treatment with navitoclax improved outcome following MI, mice from each group were then subjected to ligation of the left anterior descending coronary artery (LAD), which mimics the pathophysiology of MI (Redgrave et al., 2017).

**Figure 2:**
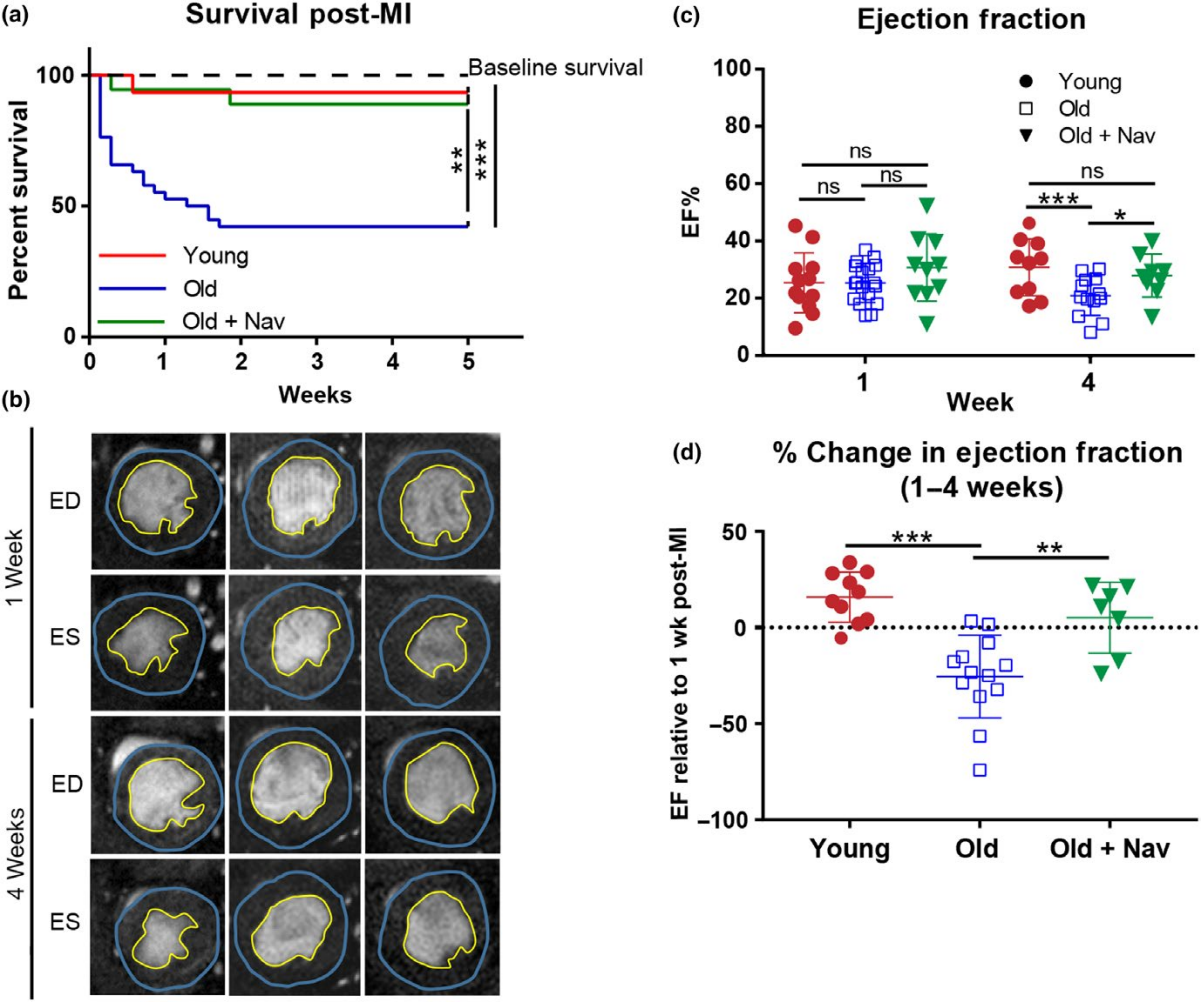
Navitoclax treatment improves survival and functional outcome following MI. (a) Kaplan–Meier survival curve following MI, *n* = 20–45 per group. Dotted line indicates survival between the age of 104–109 weeks in a cohort of vehicle‐treated mice that received no MI (*n* = 9). (b) Examples of individual short axis cine‐MR images post‐MI. ED = end diastole, ES = end systole. (c) Ejection fraction at 1 and 4 weeks post‐MI, *n* = 9–15 per group. (d) Relative change in ejection fraction between 1 and 4 weeks post‐MI, *n* = 7–11 per group. Data are mean ± *SEM*, ***p < 0.001; **p < 0.01; * *p* < 0.05 using one‐way ANOVA

We observed that aged mice had significantly higher mortality rates following MI (60% over 5 weeks) compared with young mice and that this outcome was rescued by prior navitoclax treatment (Figure 2a). This high mortality also contrasts with the low mortality observed in a cohort of aged mice that received no MI (Figure 2a) and our previously published data that demonstrated a low mortality in male C57BL/6 mice between the same ages of 104–109 weeks (Jurk et al., 2014). Together these data suggest that reduced survival in old mice following LAD‐ligation was a result of the MI and was not due to aging alone. No difference in EF was observed between the MI groups at 1 week. However, in contrast to young mice, old mice show a significant reduction in EF between 1 and 4 weeks post‐MI. Importantly, navitoclax was able to rescue this functional decline (Figure 2b–d) which may help to explain the improved survival of this group. Furthermore, expression of senescence markers p16 and p21 at 4 weeks following MI was reduced in the hearts of navitoclax‐treated mice, consistent with reduction of the senescence burden (supporting information Figure S1A).

Collectively, this study shows that pharmacological clearance of senescent cells in aging mice alleviates age‐dependent myocardial remodelling and attenuates expression of profibrotic mediators. Navitoclax improved the maintenance of cardiac function following MI, ultimately increasing survival. An important limitation of this study is that our experimental strategy was not able to distinguish which senescent cell types are responsible for this effect, and it is possible that clearance of senescent cells in noncardiac organs impact on survival following MI. We have focussed our attention on CMs in this study as our earlier findings showed that, in the heart, markers of senescence accumulate primarily in CMs during aging (Anderson et al., 2019). However, further studies using animal models where senescent cells can be cleared in a cell‐type specific manner are required to formally show the contribution of senescent CMs to cardiac recovery post‐MI. As senolytics have now moved into clinical trials in other fibrotic diseases (Justice et al., 2019), we propose that senolytics may help to reduce the morbidity and mortality associated with MI in the older population.

## CONFLICT OF INTEREST

None Declared.

## AUTHOR CONTRIBUTIONS

AW, ED, RR, SV and ST‐C performed experiments. AO, HMA and IS contributed to supervision. GDR and JFP designed and supervised the study. GDR, JFP and HMA wrote the manuscript with contributions from all authors.
