# Supplementary material for: Pharmacological clearance of senescent cells improves survival and recovery in aged mice following acute myocardial infarction
Source: Aging Cell. 2019 Mar 28;18(3):e12945. doi: 10.1111/acel.12945 (PMC6516151; doi:10.1111/acel.12945)
Supplement: Supplementary file 1 [file ACEL-18-e12945-s001.docx]

**
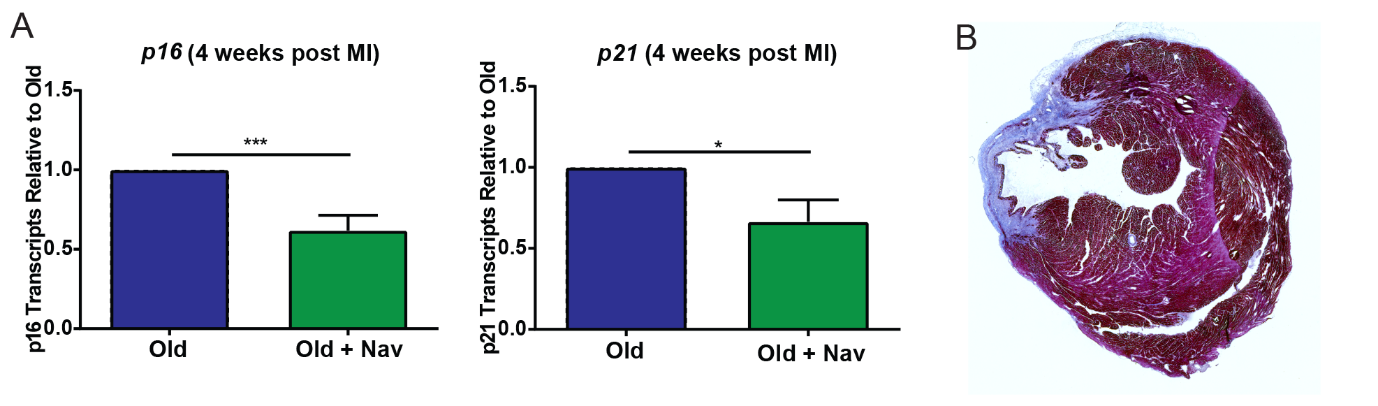
**

**Supplementary Figure 1 A)** Relative expression of p16 and p21 in whole digests of the hearts of vehicle and navitoclax treated aged mice at 5 weeks post LAD-ligation quantified by qRT-PCR. Data are mean±SEM of n>7 mice. **B)** Example of Masson's trichrome staining demonstrating a transmural infarct at 4 weeks post-Ligation. For all figures asterisks denote a statistical significance ***P<0.001, *P<0.05, using a student’s T-test.
