# Supplementary material for: Pharmacological clearance of senescent cells improves survival and recovery in aged mice following acute myocardial infarction
Source: Aging Cell. 2019 Mar 28;18(3):e12945. doi: 10.1111/acel.12945 (PMC6516151; doi:10.1111/acel.12945)
Supplement: Supplementary file 2 [file ACEL-18-e12945-s002.docx]

**Pharmacological clearance of senescent cells improves survival and recovery in aged mice following acute myocardial infarction.**

Anna Walaszczyk^1^, Emily Dookun^1^, Rachael Redgrave^1^, Simon Tual-Chalot^1^, Stella Victorelli^2,3^ Ioakim Spyridopoulos^1^, Andrew Owens^1^, Helen M. Arthur^1^, [João F. Passos](http://www.ncbi.nlm.nih.gov/pubmed/?term=Passos%20JF%5Bauth%5D)^2,3^, Gavin D Richardson^1.^

^1^Cardiovascular Research Centre, Institute of Genetic Medicine, Newcastle University, Newcastle upon Tyne, UK.

^2^Institute for Cell and Molecular Biosciences Newcastle University, Newcastle upon Tyne, UK.

^3^ Department of Physiology and Biomedical Engineering, Mayo Clinic, Rochester, MN, USA.

**Corresponding authors:**

Dr Gavin D. Richardson, Cardiovascular Research Centre, Institute of Genetic Medicine, Newcastle University, Newcastle upon Tyne, NE1 3BZ UK, Telephone: +44 (0)191 241 8615 Fax: +44 (0)191 241 8666 Email [Gavin.Richardson@ncl.ac.uk](mailto:Gavin.Richardson@ncl.ac.uk)

Dr João Passos, Department of Physiology and Biomedical Engineering, Mayo Clinic, Rochester, MN, USA. Telephone: 507-293-9785, Email: [Passos.Joao@mayo.edu](mailto:Passos.Joao@mayo.edu).

**EXPERIMENTAL PROCEDURES**

**Animals, Procedures and Senolytic Treatment.**

Mice were purchased from Charles River (Charles River Laboratories International, UK). The project was approved by the Faculty of Medical Sciences Ethical Review Committee, Newcastle University and the UK home office. Male C57BL/6 mice were used at either 13 weeks (3-months) or 100 weeks (23-months) of age. 100 week old mice were randomly assigned to a treatment group and ABT263 (navitoclax) or vehicle alone. Navitoclax was administered by gavage at 50 mg/kg body weight per day (mg/kg/d) for 7 d per cycle for two cycles with a 1-week interval between the cycles (Fig 1A). Mice from each experimental group were then randomly assigned to two cohorts 1) Mice were culled by humane methods and the hearts were collected directly into 50 mM KCl, to arrest in diastole, and embedded in paraffin wax. 2) Mice were subject to ligation of the left anterior descending artery (LAD), a model of Myocardial infarction.

**Mouse model of myocardial infarction.**

Myocardial infarction was induced using the surgical LAD-ligation model as described previously (Redgrave *et al.* 2016). All procedures were conducted in accordance with the Guidance on the Operation of the Animals (Scientific Procedures) Act, 1986 (UK Home Office) and approved by the local ethics committee. Mice were pre-medicated with fentanyl/fluanisone (‘Hypnorm’, 0.4 ml/kg) for intra-operative analgesia and anaesthetised was achieved using isoflurane (3% isoflurane/97% oxygen). Anaesthesia was maintained using mechanical ventilation (Inspira Ventilator) following endotracheal intubation using 130–140 breaths per minute and 5 ml/kg tidal volume initially, increased to 7.5 ml/kg post-thoracotomy. Left-side thoracotomy was performed via the fourth intercostal space and the left anterior descending (LAD) coronary artery was ligated directly below the left atria with a 7-0 prolene suture. The success of vessel occlusion was verified by blanching in the left ventricular myocardium. Following the closure of the chest mice were extubated and post-operative analgesia (buprenorphine Vetergesic, 0.05 mg/kg) was administered subcutaneously. Mice were allowed to recover in a 33 °C incubator for 2 hr post-surgery and provided with soaked diet.

**RNA in situ Hybridization**.

RNA-ISH was performed using the RNAscope kit from Advanced Cell Diagnostics Inc. (ACD, Newark, USA) and the cdkn2a (p16) probe: Mm-Cdkn2a-tv2 Ref 447491, as per the manufactures instructions. Briefly, paraffin sections were deparaffinised with Histoclear, rehydrated in graded ethanol (EtOH), and H_2_O_2_ was applied for 10 min at RT followed by two washes in H_2_O. Sections were placed in hot retrieval reagent and heated for 30 min. After washes in H_2_O and 100% EtOH, sections were air dried. Sections were treated with protease plus for 30 min at 40 ºC, washed with H_2_O, and incubated with target probe for 2 hr at 40 ºC. Afterwards, slides were washed with H_2_O followed by incubation with AMP1 (30 min at 40 ºC) and next washed with wash buffer (WB) and AMP2 (15 min at 40 ºC), WB and AMP3 (30 min at 40 ºC), WB and AMP4 (15 min at 40 ºC), WB6 and AMP5 (30 min at RT) and WB and, finally, AMP6 (15 min at RT). Finally, RNAscope 2.5 HD Reagent kit-RED was used for chromogenic labelling. After counterstaining with haematoxylin, sections were mounted and coverslipped.

### Histology and Immunohistochemistry.

Deparaffinization, hydration, and antigen retrieval of formalin-fixed paraffin-embedded heart tissues was performed as previously described (Richardson *et al.* 2015). Sections were incubated with 5% BSA/PBS. After washing primary antibody was diluted in 2% BSA/PBS and incubated overnight at 4°C. After 2 × 5 min PBS washes, sections were incubated in secondary antibody in 2% BSA/PBS and incubated for 1 hour at room temperature. Slides were mounted in VECTASHIELD with DAPI. For CM size analysis after cardiomyocyte staining slides were additionally incubated for 1 hour at room temperature with WGA (674nm). Primary antibodies used: goat Poly-clonal anti-troponin C (ab30807, Abcam, Cambridge, UK), Rat anti-TGFβ2 (MAB73461, R&D Systems, Minneapolis, MN, USA ) Secondary antibody used: Alexa Fluor® 488, Alexa Fluor® 594, or Alexa Fluor® 647 (Invitrogen, Waltham, MA, USA). For Trichrome (Masson) stain deparaffinized slides were incubated overnight with Bouin’s solution, tap water washed, stained with Weigert’s iron hematoxylin solution, washed. Briebrich Scarlet acid fuchsin solution (5 min), washed, incubated for 10 min in Phosphomolybdic-Phosphotungstic Acid Solution, drain, aniline blue (5 min), washed, incubation in 1% acetic acid (1 min). Washed slides were dehydrated and mounted in histomount.

**Magnetic resonance Imaging and Analysis.**

Magnetic resonance images were acquired using a horizontal bore 7.0T Varian microimaging system (Varian Inc., Palo Alto, CA, USA) equipped with a 12-cm microimaging gradient insert (40 gauss/cm) and analysed as described previously (Redgrave *et al.* 2017). Percentage change in wall thickening was calculated from the mean of wall thickness at 3 points in all slices at end systole and end diastole thickness. Percentage change=(EST-EDT)/EDTx100 as previously (Anderson *et al.* 2019). All analysis was performed in a blinded manner.

**Post AMI Survival.**

Mice that died during surgery or within 24 hrs post-operatively were considered to have died as a direct result of the surgical procedure and excluded from studies. Mice were monitored during for 5-weeks post-LAD and humanely killed when in a moribund state (as assessed in a blinded fashion by a Qualified Named Animal Care and Welfare Officer as well as a Named Veterinary Surgeon). Survival was assessed using the Kaplan–Meier estimator statistical test. Cardiac tissues where collected from all mice and stained with Masson’s trichrome to confirm that every mouse in the study had received a permanent ligation of the LAD **(Fig S1B)**.

### Real Time PCR (qRT-PCR)

### Qiagen RNA extraction kit was used of RNA isolation. First Strand cDNA Synthesis Kit (Thermo Fisher Scientific, Waltham, MA, US) was used for cDNA synthesis. Real-time PCR was performed in a 7500 Fast Real Time PCR System using Platinum SYBR Green qPCR Super Mix-UDG (Invitrogen) with p16 forward: 5’-GCAGTTCGAATCTGCACCG p16 reverse: 5’-GTGTGCATGACGTGCGGG and p21 forward: 5’-CCTGGTGATGTCCGACCTG, p21 reverse: 5’-CCATGAGCGCATCGCAATC. Target gene expression was calculated by the comparative CT method and normalized to the expression of 18S rRNA (forward 5’-CGGCTACCACATCCAAGGAA reverse: 5’-AGCCGCGGTAATTCCAGC).

### Statistical Analysis. We conducted two-tailed *t*-tests, one-way ANOVA and Student T- tests using GraphPad Prism (GraphPad Software, San Diego, CA, USA).

Anderson R, Lagnado A, Maggiorani D, Walaszczyk A, Dookun E, Chapman J, Birch J, Salmonowicz H, Ogrodnik M, Jurk D, Proctor C, Correia-Melo C, Victorelli S, Fielder E, Berlinguer-Palmini R, Owens WA, Greaves L, Kolsky K, Parini A, Douin-Echinard V, LeBrasseur N, Arthur H, Tual-Chalot S, Schafer M, Roos C, Miller J, Robertson N, Mann J, Adams PD, Tchkonia T, Kirkland JL, Mialet-Perez J, Richardson GD, Passos JF (2019). Length-independent telomere damage drives cardiomyocyte senescence. *EMBO J*. doi: 10.15252/embj.2018100492.

Redgrave RE, Tual-Chalot S, Davison BJ, Greally E, Santibanez-Koref M, Schneider JE, Blamire AM, Arthur HM (2016). Using MRI to predict future adverse cardiac remodelling in a male mouse model of myocardial infarction. *Int J Cardiol Heart Vasc*. **11**, 29-34.

Redgrave RE, Tual-Chalot S, Davison BJ, Singh E, Hall D, Amirrasouli MM, Gilchrist D, Medvinsky A, Arthur HM (2017). Cardiosphere-Derived Cells Require Endoglin for Paracrine-Mediated Angiogenesis. *Stem Cell Reports*. **8**, 1287-1298.

Richardson GD, Laval S, Owens WA (2015). Cardiomyocyte Regeneration in the mdx Mouse Model of Nonischemic Cardiomyopathy. *Stem Cells Dev*. **24**, 1672-1679.
